# Leptospirosis diagnosis among patients suspected of dengue fever in Brazil

**DOI:** 10.1590/1678-9199-JVATITD-2020-0118

**Published:** 2021-03-26

**Authors:** Felipe Fornazari, Virgínia Bodelão Richini-Pereira, Sâmea Fernandes Joaquim, Pedro Gabriel Nachtigall, Helio Langoni

**Affiliations:** 1 Department of Animal Production and Preventive Veterinary Medicine, School of Veterinary Medicine and Animal Husbandry, São Paulo State University (UNESP), Botucatu, SP, Brazil.; 2 Center of Regional Laboratory II Bauru, Adolfo Lutz Institute, Bauru, SP, Brazil.; 3 Laboratory of Applied Toxinology, Butantan Institute, São Paulo, SP, Brazil.

**Keywords:** Leptospira, Microscopic agglutination test, PCR, Unreported disease, Zoonosis

## Abstract

**Background:**

The early symptoms of leptospirosis and dengue fever are difficult to distinguish and can cause diagnostic confusion. Due to the large dengue epidemics that has occurred in Brazil in recent years, it is possible that cases of leptospirosis were unreported. Therefore, we performed a retrospective study to detect leptospirosis in patients who were tested for dengue, but whose laboratory diagnoses were negative.

**Methods:**

Sera samples from 2,017 patients from 48 cities located in the central region of São Paulo state, Brazil, were studied. All samples were subjected to the microscopic agglutination test (MAT), 305 of which were taken from patients five days or less since the onset of symptoms, and were additionally subjected to real-time polymerase chain reaction (PCR).

**Results:**

The overall prevalence of leptospirosis cases was 21 (1.04%), with 20 through MAT (18 for Icterohaemorrhagiae and two for the Cynopteri serogroup) and one through PCR (amplicon sequencing compatible with *Leptospira interrogans*). According to previously established criteria, eight cases of leptospirosis were classified as “confirmed” and 13 as “probable”. The Brazilian notification system for health surveillance had no records for 16 patients positive for leptospirosis and, thus, they were considered unreported cases. Statistical analyses revealed that the prevalence of leptospirosis was higher in men (1.56%) than in women (0.56%), and the mean age was higher in positive patients (43.7 years) than in negative ones (32.3 years).

**Conclusion:**

The results indicated that patients suspected of dengue fever had evidence of leptospirosis or *Leptospira* infection, and most of these cases were unreported in the Brazilian notification system. The high burden of dengue may contribute to the misdiagnosis of leptospirosis, and health professionals should increase their awareness of leptospirosis as an important differential diagnosis of patients with suspicion of dengue.

## Background

Leptospirosis is among the leading zoonotic causes of morbidity and mortality worldwide, with annual estimates of 1.03 million cases and 59,900 deaths [1]. In tropical areas, leptospirosis can be indistinguishable from other febrile illnesses, such as dengue fever [2]. Early-phase leptospirosis and classic dengue have unspecific and overlapping symptoms, including fever, headache, and muscle pain, which can lead to delayed or missed diagnosis of leptospirosis. This confusion between the early symptoms of both diseases can contribute to delayed diagnosis of leptospirosis cases, which leads to higher mortality rates [3]. Few studies have been performed in Brazil comparing dengue and leptospirosis diagnosis. In Fortaleza state, approximately 20% of dengue-like cases may be leptospirosis cases in areas where the two diseases are endemic; and the actual number of leptospirosis cases may be 29 to 49 times higher than those diagnosed and reported by the health services [4].

Brazil has experienced a large epidemic of dengue in recent years, reaching a high incidence between 2015 and 2016, when more than three million cases were estimated [5]. During this period, it is possible that many patients with leptospirosis were misdiagnosed due to the greater attention directed toward dengue. Another aggravating factor is that leptospirosis is a highly neglected disease in Brazil, affecting marginalized populations marked by poverty, low education levels, and racial segregation [6]. National tenders do not make mentions of research or intervention investments for this disease [6], and the Brazilian neglected disease program does not include leptospirosis as a priority area [7]. Thus, physicians and other healthcare professional may overlook leptospirosis more often than other febrile illnesses.

The aim of the present study is to investigate whether leptospirosis was the cause of illness in patients with suspected dengue who yielded negative results in laboratory tests. In addition, unreport of leptospirosis cases was investigated in the Brazilian notification system.

## Methods

### Study design

**Figure 1:**
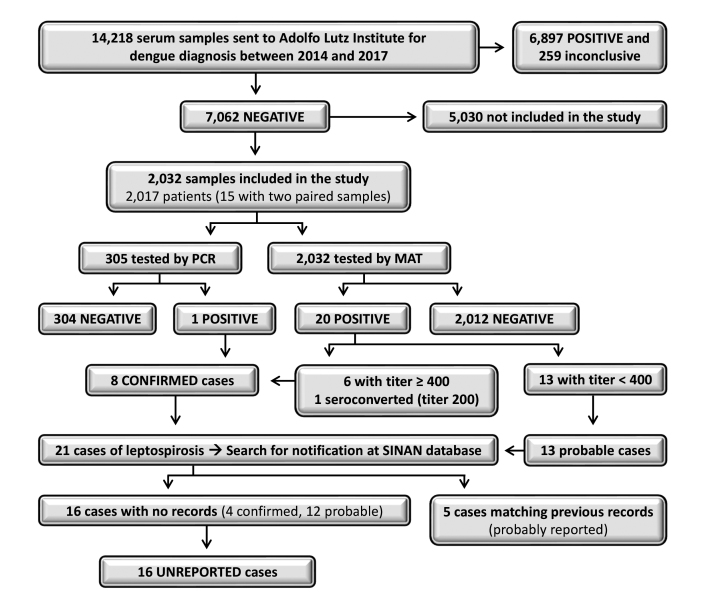
Flowchart of the study design, methodology, and results of leptospirosis diagnosis among patients suspected of dengue fever in São Paulo state, Brazil.

A retrospective survey was conducted using serum samples stored (-80°C) at the Adolfo Lutz Institute (IAL), Regional Laboratory Center II Bauru, São Paulo state (SP), Brazil, which performs laboratory diagnosis for healthcare units from 38 neighboring cities. The cities with the largest number of studied samples (more than 100) were Agudos, Barra Bonita, Bauru, Igaraçu do Tietê, Jaú and Lins (Additional file 1), which together comprises a total population of 707,957 habitants [8]. Samples of patients tested for dengue between 2014 and 2017 with negative results were included in the study. Dengue diagnosis was performed using enzyme-linked immunosorbent assays (Panbio^®^ Dengue Early ELISA or Panbio^®^ Dengue IgM Capture ELISA). Most patients tested in the IAL were from the central region of SP, where leptospirosis reporting in the majority of cities were below six cases during the study period (Additional file 2). The study design is shown in Figure 1.

### Patients and samples

Among 14,218 samples that were tested for dengue in the intended period, we randomly selected 2,032 belonging to 2,017 patients (15 patients had acute and convalescent samples) (Figure 1). The minimum sample size was estimated as 1,367 patients considering an anticipated prevalence of 3.7% [9], 1% confidence limit, and a population of one million. The OpenEpi platform was used for the statistical calculation [10].

### Diagnosis of leptospirosis

Serological diagnosis of leptospirosis was performed in all samples using a microscopic agglutination test (MAT) executed with standard protocols [11]. Five reference strains, representing the major *Leptospira* serogroups, were initially selected according to their good performance in identifying MAT-confirmed cases of leptospirosis in previous studies conducted in Brazil [12]. The antigens were kindly provided by the Oswaldo Cruz Foundation (Salvador, Brazil), and included the following serovars and strains: Copenhageni (M20), Canicola (Hond Utrecht IV), Autumnalis (Akyiami), Ballum (Mus 127) and Cynopteri (3522 C). An additional strain named RGA (Icterohaemorrhagiae serogroup) was included due to its good performance in our laboratory tests. The maintenance of the serovar Ballum in the culture media used in our laboratory was extremely difficult, thus, we later excluded this antigen from the study.

Molecular diagnosis was performed in samples from 305 patients who had five days or less between the onset of symptoms and blood collection, which is the period with higher chances of detecting leptospires in blood [13]. Serum DNA was extracted using an Illustra^TM^ Blood Genomic Prep Mini spin kit according to the manufacturer’s instructions. To optimize the concentration of the assay template, DNA extraction was performed with 400 µL of serum instead of 200 µL. Samples were subjected to real-time polymerase chain reaction (PCR) using StepOne^TM^ Plus Real Time PCR System equipment, Power SYBR^®^ Green mix, and Lipl32 primers [14]. Cycling conditions consisted of initial denaturation at 95˚C for 10 min, followed by 45 cycles of amplification (95˚C for 10 s and 58˚C for 30 min) and melting curve analysis. PCR amplicons were sequenced using the Sanger method as previously described [15] and compared to the *Leptospira* sequences available from the NCBI collection.

Since early symptoms of dengue and leptospirosis are very similar and difficult to distinguish [2, 3], we considered that patients tested for dengue had clinical signs consistent with leptospirosis. Thus, positive cases in leptospirosis diagnosis were classified as “probable” or “confirmed” according to criteria established by the Leptospirosis Burden Epidemiology Reference Group. Probable cases were defined when MAT titre were 100 or 200 in single samples, while confirmed cases were defined in any one of the following: MAT titre ≥ 400; fourfold increase in MAT titre in acute and convalescent samples; detection of pathogenic *Leptospira* DNA by PCR [16].

### Unreported leptospirosis cases

Leptospirosis cases were verified if they were unreported by accessing the Notifiable Diseases Information System database (SINAN) [17]. Since the incidence of reported leptospirosis was below six cases in most of the studied cities (Additional file 2), notification of each case was possible by searching for records using the variables “city” and “year of diagnosis”. If one or more cases were registered with the same data, the variables “age”, “gender”, and “month of symptoms” of the investigated patient were included in the search. Leptospirosis cases with no matching records in SINAN were classified as “unreported”, and those for which all the variables matched previous records were classified as “probably reported” (Figure 1).

### Data analysis

Frequencies were calculated with a 5% significance level. The results of leptospirosis diagnosis were compared with patient’s sex using the Chi-square test and, if significance was detected, the odds ratio (OR) was calculated. The age of the patients was tested for normality of data using the Shapiro-Wilk test and, in case of non-normal distribution, comparison with the results of leptospirosis diagnosis was made through the Kruskal-Wallis test followed by the post-hoc Dunn’s test. The variables were considered significant when the P value was < 0.05. All analyses were performed using the R software.

## Results

**Figure 2:**
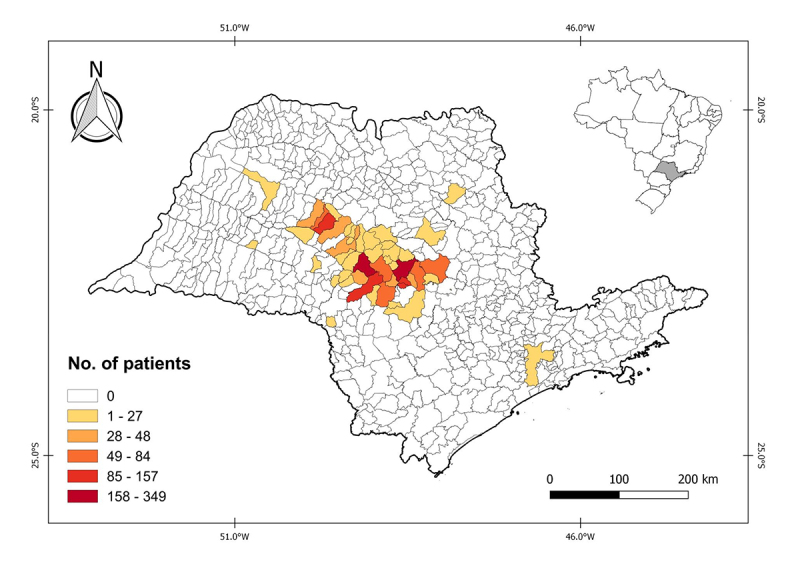
Number of patients by municipality tested for leptospirosis in the central region of São Paulo state, Brazil (the map was created using the QGIS 2.18 software with graduated style and Natural Breaks algorithm).

Subjects were from 48 municipalities located mainly in the central region of SP (Figure 2 and Additional file 1). Most patients were female (52.5%), ages ranged from six days to 88 years (mean of 32.4 years), and the time between onset of symptoms and blood sampling ranged from one to 73 days (mean of 8.4 days). The 15 patients with paired samples had a mean of 8.8 days between sample collection. 

The diagnosis of leptospirosis revealed 21 (1.04%; CI 95% 0.6 - 1.5) positive patients distributed in 11 cities, of which 20 were positive via MAT (18 for Icterohaemorrhagiae and 2 for Cynopteri) and one via PCR (Table 1 and Additional file 1). The PCR amplicon sequencing hit the expected region of the Lipl32 gene of *Leptospira interrogans* with 100% identity and a 4e-94 e-value (Additional file 3). Among the 21 positive cases, eight were classified as “confirmed” and 13 as “probable”. No records were found for 16 cases (four confirmed and 12 probable), thus, these were considered “unreported”. The remaining five cases matched previous records from SINAN and were, thus, considered “probably reported”. These results are summarized in Figure 1 and Table 1. Statistical analyses revealed that the prevalence of leptospirosis was higher in men (1.56%; OR = 2.8) than in women (0.56%), and the mean age was higher in positive patients (43.7 years) than in negative ones (32.3 years).

**Table 1:** Data from patients who tested positive for leptospirosis in the central region of São Paulo state, Brazil.

Patient code	Leptospirosis diagnosis			Sex	Age	Year of diagnosis	Days since onset of symptoms	Municipality	Classification of the case^a^
	MAT		PCR						
	Serogroup	Titre							
FG 10	Icter	200	-	F	38	2016	2	Bauru	Probable
FG 92	Icter	200	-	F	61	2015	2	Lins	Probable
FG 156^┼^	Icter	1600	-	M	38	2015	10	Agudos	Confirmed
FG 272	Icter	100	-	M	77	2015	15	Pederneiras	Probable
FG 327	Icter	400	-	M	58	2015	11	Lins	Confirmed
FG 490^┼^	Icter	400	-	M	59	2015	11	Duartina	Confirmed
FG 541	Cynopteri	400	-	F	24	2015	17	Jaú	Confirmed
FG 591	Icter	200	-	F	54	2015	Not available	Piratininga	Probable
FG 592	Icter	100	-	M	24	2015	7	Araçatuba	Probable
FG 1038	Icter	400	-	M	45	2014	6	Bauru	Confirmed
FG 1304	Icter	200	-	M	28	2016	10	Dois Córregos	Probable
FG 1344	Icter	200	-	M	63	2016	21	Jaú	Probable
FG 1349	Icter	200	-	M	56	2016	6	Lençóis Paulista	Probable
FG 1440	Icter	100	-	M	12	2016	15	Torrinha	Probable
FG 1453^┼^	-	-	+	M	39	2016	5	Barra Bonita	Confirmed
FG 1470^‡^	Icter	200	-	M	36	2016	9	Piratininga	Confirmed
FG 1478	Cynopteri	200	-	M	72	2016	8	Jaú	Probable
FG 1626^┼^	Icter	200	-	M	36	2016	7	Jaú	Probable
FG 1693^┼^	Icter	800	-	M	16	2016	7	Bauru	Confirmed
FG 1898	Icter	200	-	F	51	2016	7	Jaú	Probable
FG 2268	Icter	100	-	F	32	2017	14	Agudos	Probable

^┼^ Patients that matched SINAN records and were considered as “probably reported”. Outcomes of these patients were: recovery (FG156, FG1453, and FG1693), death by leptospirosis (FG1626), and death by another cause (FG490). The remaining patients did not match records from SINAN database and their clinical outcomes were not possible to obtain. ^‡^First sample was seronegative and second one seropositive (4 days later). Icter: Icterohaemorrhagiae. -: Negative. +: Positive. ^a^Classification of the leptospirosis cases as “confirmed” or “probable” according to criteria of the Leptospirosis Burden Epidemiology Reference Group

## Discussion

Leptospirosis diagnosis was confirmed among patients suspected of dengue fever in the central region of SP. Half of the confirmed cases and almost all probable cases were not recorded in the SINAN database, indicating misdiagnosis and/or misreporting. The early symptoms of dengue and leptospirosis are difficult to distinguish and are the most likely origin of diagnostic confusion, as discussed in previous studies [2, 3, 18]. The dengue epidemics in Brazil have possibly contributed to misdiagnosis, since health professionals were more prone to suspect dengue. Another aggravating factor may be the low familiarity and awareness of physicians with leptospirosis in the studied region. 

SP is the most developed and populous state of Brazil, possessing broad access to free public health services. Therefore, one would expect high efficiency in detecting and reporting diseases whose notification is mandatory. However, the results suggest that leptospirosis remains unreported even in the most developed state of Brazil. Considering the social and economic status of other Brazilian states, mainly those located in the North and Northeast regions, we strongly believe that they have higher rates of unreported leptospirosis than SP. Infectious diseases are considered particularly prone to underestimation due to their specific characteristics, such as asymptomatic or self-limiting course. Therefore, they are represented inadequately by raw surveillance data [19], corroborating our results. In order to improve the measurement of a specific disease and promote the best use of resources, public health surveillance systems should be evaluated periodically. Such improvement should assess a broad system of attributes, such as data quality, representativeness, flexibility, sensitivity, timeliness, among others [20].

The number of leptospirosis cases in the population studied was probably higher than that detected in our study. Although MAT is considered the immunological reference standard for the diagnosis of leptospirosis [21], it has several limitations, and its performance largely depends on the availability of local strains [22]. We were not able to isolate or obtain isolated strains of leptospires from humans in the studied region, and this may have limited the efficiency of our serological tests. Regarding the molecular diagnosis, it is possible that the use of sera instead of blood may have affected the PCR detection rate, since leptospires may adhere to leucocytes and/or red blood cells and incorporate into the clot [14]. However, serum may be better than whole blood as a specimen type for the molecular diagnosis of acute leptospirosis, possibly because blood contains more PCR inhibitors [23]. It is uncertain whether the type of sample affected our molecular diagnosis. Other flaws that may have limited the detection of leptospirosis were the low availability of patients with acute and convalescent samples, and the failure to employ the Ballum serovar.

Icterohaemorrhagiae serogroup prevailed among MAT-positive patients. An important retrospective study conducted in SP found that 54.8% of 9,335 seropositive patients reacted to the Icterohaemorrhagiae serogroup [24], corroborating our findings. In humans, severe leptospirosis is frequently but not invariably caused by serovars of the Icterohaemorrhagiae serogroup [13], making it one of the most notorious leptospires important to public health. Serovar Icterohaemorrhagiae is the most prevalent serovar reported in rats [25], indicating that this animal group was the possible origin of infection among the MAT-positive patients. Cynopteri was the only reactive serovar of two patients and, curiously, they were from the same city and their onset of symptoms differed by only 72 days (data not shown). According to these patients’ addresses, the distance between their houses was only 1.1 km. Since this was a retrospective study, we could not obtain more detailed information on these two patients, but their close date of symptoms and residences suggest a common source of infection by the Cynopteri serogroup. Data on the importance of Cynopteri causing leptospirosis is very scarce in Brazil, with only one well-documented case [26].

Men had a higher prevalence than women, which is in accordance with most leptospirosis studies, reflecting higher exposure levels to leptospires due to occupational and outdoor activities [27, 28]. The age of positive patients was higher than that of negative ones, suggesting greater risks of infection in individuals above 40 years of age. Age-specific behaviors can be related to *Leptospira* exposure [28]; however, high incidences are observed in many age groups above 20 years of age [16].

## Conclusions

In conclusion, approximately 1% of patients suspected of dengue fever had evidence of *Leptospira* infection, with most of these cases being unreported to the Brazilian notification system. This may imply underreporting leptospirosis in the study area. Health professionals should increase their awareness of leptospirosis among patients presenting unspecific symptoms compatible with dengue, especially during periods with a high burden of dengue. 

### Abbreviations

CI: confidence interval; DNA: deoxyribonucleic acid; ELISA: enzyme-linked immunosorbent assay; IAL: Adolfo Lutz Institute; MAT: microscopic agglutination test; OR: odds ratio; PCR: real-time polymerase chain reaction; SINAN: Notifiable Diseases Information System; SP: São Paulo state.

## Supplementary material

The following online material is available for this article:

Additional file 1. Leptospirosis diagnosis among patients suspected of dengue fever in Brazil. Number of patients tested and positive results for leptospirosis diagnosis according to the municipality of origin in the central region of São Paulo state, Brazil.

Additional file 2. Number of leptospirosis cases reported to the Notifiable Diseases Information System (SINAN) between 2014 and 2017 in São Paulo state (SP), Brazil. High reporting rates were concentrated mostly in the central-east and the southeast regions of the state. The central region of SP, where the study was conducted, presented less than six cases in most cities. Data were obtained from www.portalsinan.saude.gov in May 2020. The map was created in the QGIS 2.18 software using graduated style with Natural Breaks algorithm.

Additional file 3. Multiple sequence alignment of the PCR amplicon (FG1453) with 10 best hits obtained in the BLAST search. The consensus sequence and the identity percentage are at the top of the figure. The sequencing chromatogram is above the amplicon sequence. The NCBI accession numbers of the BLAST search are at the left side indicated by the positions 2 to 11 in the alignment. The sequences for the alignment were obtained from www.ncbi.nlm.nih.gov, and the figure was created in the Geneious v2019.2.1 software.
