# Supplementary material for: Leptospirosis diagnosis among patients suspected of dengue fever in Brazil
Source: J Venom Anim Toxins Incl Trop Dis. 2021 Mar 26;27:e20200118. doi: 10.1590/1678-9199-JVATITD-2020-0118 (PMC7996315; doi:10.1590/1678-9199-JVATITD-2020-0118)
Supplement: Additional file 1. [file 1678-9199-jvatitd-27-e20200118-s1.pdf]

J Venom Anim Toxins incl Trop Dis
https://doi.org/10.1590/1678-9199-JVATITD-2020-0118

Supplementary Material to "Leptospirosis diagnosis among patients suspected of dengue fever in Brazil"

Additional file 1. Leptospirosis diagnosis among patients suspected of dengue fever in Brazil. Number of patients tested and positive results for leptospirosis diagnosis according to the municipality of origin in the central region of São Paulo state, Brazil.

Municipality name	Number of patients	
	Tested	Positive (test)
Águas de São Pedro	1	0
Agudos	114	2 (MAT)
Araçatuba	1	1 (MAT)
Araraquara	1	0
Arealva	26	0
Avaí	3	0
Balbinos	14	0
Bariri	27	0
Barra Bonita	110	1 (PCR)
Bastos	1	0
Bauru	349	3 (MAT)
Bocaina	17	0
Boracéia	25	0
Borebi	6	0
Botucatu	1	0
Brotas	84	0
Cabrália Paulista	6	0
Cafelândia	43	0
Dois Córregos	55	1 (MAT)
Duartina	15	1 (MAT)
Getulina	9	0
Guaiçara	51	0
Iacanga	23	0
Ibitinga	1	0

Municipality name	Number of patients	
	**Tested**	**Positive (test)**
Igaraçu do Tietê	129	0
Ipaussu	1	0
Itajú	8	0
Itapuí	22	0
Jaú	292	5 (MAT)
Lençóis Paulista	68	1 (MAT)
Lins	157	2 (MAT)
Lucianópolis	17	0
Macatuba	48	0
Mineiros do Tietê	37	0
Pederneiras	54	1 (MAT)
Pirajuí	40	0
Piratininga	37	2 (MAT)
Pongaí	6	0
Pratânia	1	0
Presidente Alves	18	0
Promissão	40	0
Reginópolis	15	0
Ribeirão Preto	1	0
Sabino	15	0
São Paulo	3	0
Torrinha	14	1 (MAT)
Uru	10	0
Vera Cruz	1	0
**Total**	**2017**	**21**

MAT: microscopic agglutination test
