# Supplementary material for: Leptospirosis diagnosis among patients suspected of dengue fever in Brazil
Source: J Venom Anim Toxins Incl Trop Dis. 2021 Mar 26;27:e20200118. doi: 10.1590/1678-9199-JVATITD-2020-0118 (PMC7996315; doi:10.1590/1678-9199-JVATITD-2020-0118)
Supplement: Additional file 2. [file 1678-9199-jvatitd-27-e20200118-s2.pdf]

J Venom Anim Toxins incl Trop Dis

https://doi.org/10.1590/1678-9199-JVATITD-2020-0118

Supplementary Material to "Leptospirosis diagnosis among patients suspected of dengue fever in Brazil"

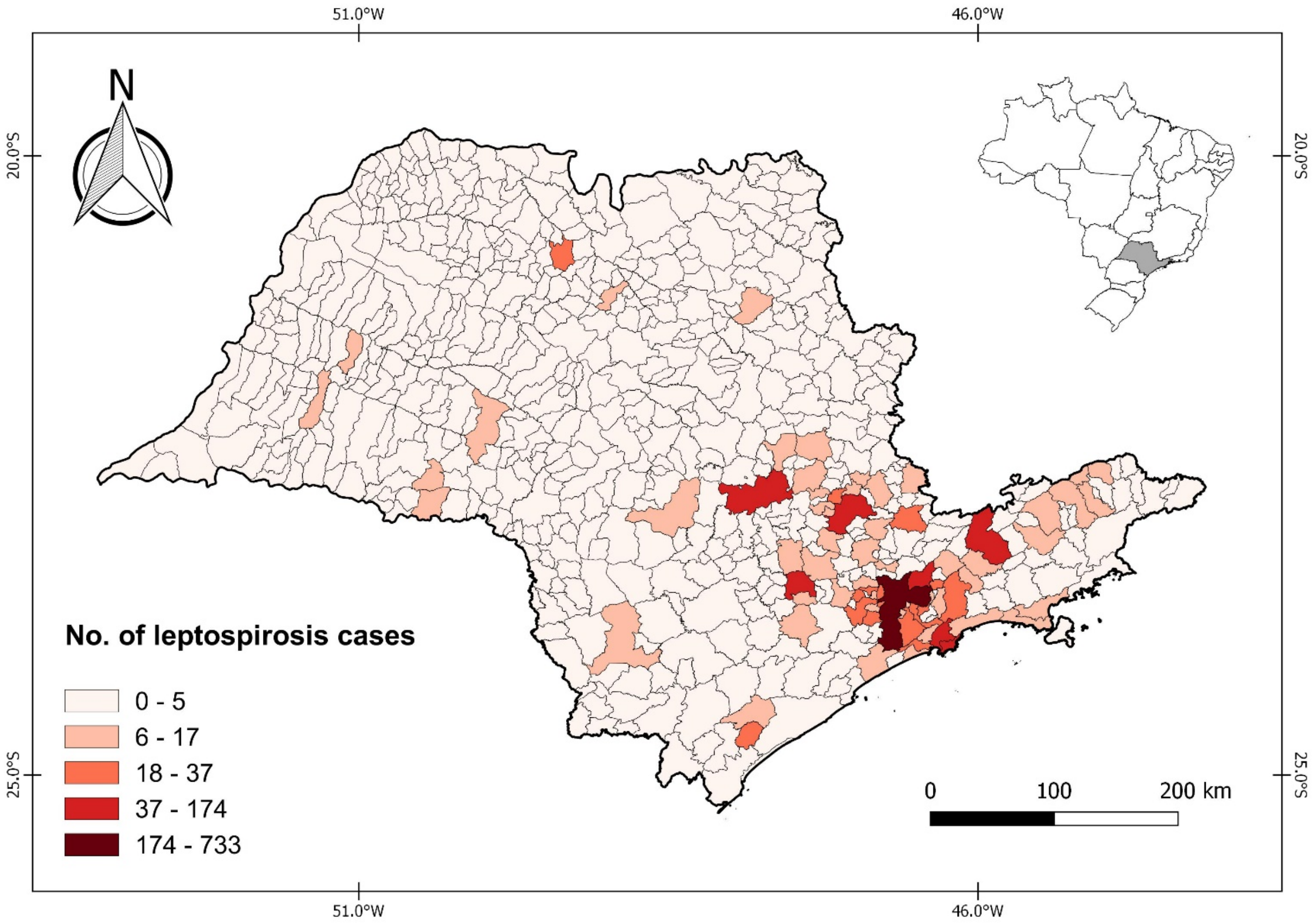

Additional file 2. Number of leptospirosis cases reported to the Notifiable Diseases Information System (SINAN) between 2014 and 2017 in São Paulo state (SP), Brazil. High reporting rates were concentrated mostly in the central-east and the southeast regions of the state. The central region of SP, where the study was conducted, presented less than six cases in most cities. Data were obtained from www.portalsinan.saude.gov in May 2020. The map was created in the QGIS 2.18 software using graduated style with Natural Breaks algorithm.
