# Supplementary material for: Leptospirosis diagnosis among patients suspected of dengue fever in Brazil
Source: J Venom Anim Toxins Incl Trop Dis. 2021 Mar 26;27:e20200118. doi: 10.1590/1678-9199-JVATITD-2020-0118 (PMC7996315; doi:10.1590/1678-9199-JVATITD-2020-0118)
Supplement: Additional file 3. [file 1678-9199-jvatitd-27-e20200118-s3.pdf]

J Venom Anim Toxins incl Trop Dis

https://doi.org/10.1590/1678-9199-JVATITD-2020-0118

Supplementary Material to “Leptospirosis diagnosis among patients suspected of dengue fever in Brazil”

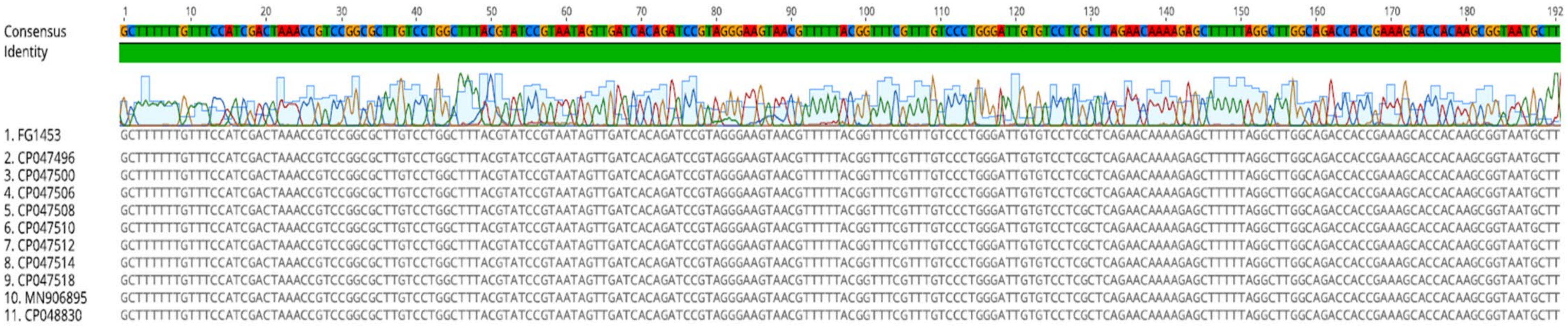

Additional file 3. Multiple sequence alignment of the PCR amplicon (FG1453) with 10 best hits obtained in the BLAST search. The consensus sequence and the identity percentage are at the top of the figure. The sequencing chromatogram is above the amplicon sequence. The NCBI accession numbers of the BLAST search are at the left side indicated by the positions 2 to 11 in the alignment. The sequences for the alignment were obtained from www.ncbi.nlm.nih.gov, and the figure was created in the Geneious v2019.2.1 software.
